# Supplementary material for: “We can’t do without it”: Parent and call-handler experiences of video triage of children at a medical helpline
Source: PLoS One. 2022 Apr 14;17(4):e0266007. doi: 10.1371/journal.pone.0266007 (PMC9009705; doi:10.1371/journal.pone.0266007)
Supplement: S1 Appendix — Parents’ questionnaires from the study regarding airway symptoms (Appendix 1A) and fever (Appendix 1B). CPR: Central Person Register (personal identification number). (PDF) [file pone.0266007.s001.pdf]

Appendix 1A+B. Parents' questionnaires.

1A. Questionnaire sent to all parents in the video triage study regarding airway symptoms

Dear parents

Please fill out the survey below, your opinion is of great importance to us.

Thank you!

Your child's CPR-number:

* must provide value

[ ]

When did you talk to the helpline 1813 about your sick child? (press "Today" if it was today)

[ ] Today D-M-Y

Are you overall satisfied with the call to the helpline 1813 today?

○ To a very large extent ○ To a large extent ○ To a moderate extent ○ To a small extent ○ Not at all

reset

Did you get answers to your questions after the call to the helpline 1813 today?

○ To a very large extent ○ To a large extent ○ To a moderate extent ○ To a small extent ○ Not at all

reset

Did you feel safe about the assessment of your child today?

○ To a very large extent ○ To a large extent ○ To a moderate extent ○ To a small extent ○ Not at all

reset

Did you feel safe about the plan for your child?

○ To a very large extent ○ To a large extent ○ To a moderate extent ○ To a small extent ○ Not at all

reset

Was your child assessed on video at 1813 today?

○ Yes ○ No

reset

In your opinion, should video calls be a permanent option at the helpline 1813?

○ Yes ○ No ○ I don't know

reset

You are welcome to leave a comment here:

[ ]

CPR: Central Person Register (personal identification number)

1B. Questionnaire sent to all parents in the video triage study regarding fever

Dear parent

please fill out the survey below. It's of great help to us.

Thank you!

Your child's CPR-number:

* must provide value

When did you contact the helpline 1813 about your child? (press "today" if it was today) Today D-M-Y

Are you overall satisfied with the call to the helpline 1813 today?

○ To a very large extent ○ To a large extent ○ To a moderate extent ○ To a small extent ○ Not at all

reset

Did you get answers to your questions after the call to the helpline 1813 today?

○ To a very large extent ○ To a large extent ○ To a moderate extent ○ To a small extent ○ Not at all

reset

Did you feel safe about the assessment of your child today?

○ To a very large extent ○ To a large extent ○ To a moderate extent ○ To a small extent ○ Not at all

reset

Did you feel safe about the plan for your child?

○ To a very large extent ○ To a large extent ○ To a moderate extent ○ To a small extent ○ Not at all

reset

BEFORE you had talked to the nurse at the helpline 1813?, how worried were you about the situation that you called about, on a scale from 1 to 5, where 1 is minimally worried and 5 is maximally worried?

○ 1 ○ 2 ○ 3 ○ 4 ○ 5

reset

AFTER you had talked to the nurse at the helpline 1813?, how worried were you about the situation that you called about, on a scale from 1 to 5, where 1 is minimally worried and 5 is maximally worried?

○ 1 ○ 2 ○ 3 ○ 4 ○ 5

reset

Was your child assessed on video at 1813 today?

○ Yes ○ We tried, but did not succeed due to technical problems ○ No

reset

In your opinion, should video calls be a permanent option at the helpline 1813?

○ Yes ○ No ○ I don't know

reset

You are welcome to leave a comment here:

CPR: Central Person Register (personal identification number)
