# Supplementary material for: “We can’t do without it”: Parent and call-handler experiences of video triage of children at a medical helpline
Source: PLoS One. 2022 Apr 14;17(4):e0266007. doi: 10.1371/journal.pone.0266007 (PMC9009705; doi:10.1371/journal.pone.0266007)
Supplement: S2 Appendix — Call-handlers’ questionnaires from the study regarding airway symptoms (Appendix 2A) and fever (Appendix 2B). CPR: Central Person Register (personal identification number). (PDF) [file pone.0266007.s002.pdf]

2A+B. Call-handlers' questionnaires

2A. Questionnaire to be filled out after all calls in the video triage project concerning airway symptoms. The last three questions only appeared after calls belonging to the video triage group.

Dear call-handler,

thank you for filling out the survey below, it if of great importance!

CPR-number
* must provide value

Time and date when filling out survey (press "Now"): Now D-M-Y H:M
* must provide value

Was the parent offered video call?
* must provide value

Yes, and accepted / Yes, but did not want to participate / No, it is a patient from the telephone triage group / Yes, but the video call was not succesfull

reset

What was the child's symptom?
* must provide value

Coughing/difficulty breathing / Cold / Suspected influenza / Other

reset

Are you overall satisfied with the call ?
* must provide value

To a very large extent / To a large extent / To a moderate extent / To a small extent / Not at all

reset

Was it technically easy to perform the video call?
* must provide value

To a very large extent / To a large extent / To a moderate extent / To a small extent / Not at all

reset

How was the sound- and image quality of the call?
* must provide value

Very good / God / Alright / Bad / Very bad

reset

Did you feel more reassured about the choice of response, now that you could see and hear the child, than if it had been a standard telephone call?
* must provide value

To a very large extent / To a large extent / To a moderate extent / To a small extent / Not at all

reset

You are welcome to leave a comment here:

CPR: Central Person Register (personal identification number)

2B. Questionnaire to be filled out after all calls in the video triage project concerning fever.

Dear call-handler,

please fill out the questionnaire below, it os of great importance!

1) CPR-number
* must provide value

2) Time and date when filling out the questionnaire (press "Now"):
* must provide value

Now D-M-Y H:M

3) Was the parent offered video call?
* must provide value

○ Yes, and accepted ○ Yes, but did not want to participate ○ No, it's a patient from the telephone triage group ○ Yes, but the video call was not succesful

reset

4) What was the child's symptom?
* must provide value

○ Fever without known focus ○ Fever and suspicion of meningitis ○ Fever and respiratory symptoms ○ Fever and vomiting/diarrhea ○ Fever and rash ○ Fever and stomach pain ○ Fever and urogenital symptoms ○ Fever and other symptom

reset

5) Are you overall satisfied with the call?
* must provide value

○ To a very large extent ○ To a large extent ○ To some extent ○ To a small extent ○ Not at all

reset

6) On a scale from 1 to 5, where 1 is minimally worried and 5 is maximally worried, how worried are you that the child is seriously ill?
* must provide value

○ 1 ○ 2 ○ 3 ○ 4 ○ 5

reset

7) You are welcome to leave a comment here:

CPR: Central Person Register (personal identification number)
