# Supplementary material for: “We can’t do without it”: Parent and call-handler experiences of video triage of children at a medical helpline
Source: PLoS One. 2022 Apr 14;17(4):e0266007. doi: 10.1371/journal.pone.0266007 (PMC9009705; doi:10.1371/journal.pone.0266007)
Supplement: S3 Appendix — MH1813: Medical Helpline 1813. (PDF) [file pone.0266007.s003.pdf]

Appendix 3. Interview guide, used in semi-structured interviews with call-handlers.

Demography

For how long have you been working as a nurse?

For how long have you worked at MH1813?

As a nurse, did you work with children before you started at MH1813? (How do you feel about answering calls about children?)

Video triage

How do you in general feel about video triaging?

When are video calls useful? (Why or why not? In what situations?)

Do you think that video calls change your way to triage? (Elaborate/give examples)

Do you think that your choice of response has changed because of video calls in some cases? (Give a couple of examples)

In what situations, other than we have tried in these projects, do you think video calls can be useful?

Project set-up, workflow, implementation

What works well in the projects?

What do you feel could be different about the projects?

How do you feel about your introduction to the projects?

When you receive a call that fits the inclusion criteria, do you always include it to the project? (If not, why?)

Do you always include the calls alternately to the video- and the telephone group?

What do you think about the technical solution?

Has the technical solution been a barrier to including patients?

What could motivate you to include more patients to the projects?

Perspectives

When the project is finished, do you wish that there still will be a possibility of using video calls?

When video calls are implemented for all at MH1813, do you think it is necessary that the triage tool gets updated?

It is necessary for the rest of the staff receives education about video triage, when the possibility is introduced?

Is there anything else you would like to add about the project or about video triage?

(MH1813: Medical Helpline 1813)
