# Supplementary material for: “We can’t do without it”: Parent and call-handler experiences of video triage of children at a medical helpline
Source: PLoS One. 2022 Apr 14;17(4):e0266007. doi: 10.1371/journal.pone.0266007 (PMC9009705; doi:10.1371/journal.pone.0266007)
Supplement: S4 Appendix — (PDF) [file pone.0266007.s004.pdf]

Appendix 4. Key process evaluation components (Steckler and Linnan, 2004)

**Component**	**Definition**
Context	Aspects of the larger social, political, and economic environment that may influence intervention implementation.
Reach	The proportion of intended target audience that participates in an intervention. If there are multiple interventions, then it is the proportion that participates in each intervention or component. It is often measured by attendance. Reach is a characteristic of the target audience.
Dose delivered	The number or amount of intended units of each intervention or each component delivered or provided. Dose delivered is a function of efforts of the intervention providers.
Dose received	The extent to which participants actively engage with, interact with, are receptive to, and/or use materials or recommended resources. Dose received is a characteristic of the target audience and it assesses the extent of engagement of participants with the intervention.
Fidelity	The extent to which the intervention was delivered as planned. It represents the quality and integrity of the intervention as conceived by the developers. Fidelity is a function of the intervention providers.
Implementation	A composite score that indicates the extent to which the intervention has been implemented and received by the intended audience.
Recruitment	Procedures used to approach and attract participants. Recruitment often occurs at the individual and organizational/community levels.
