# Supplementary material for: “We can’t do without it”: Parent and call-handler experiences of video triage of children at a medical helpline
Source: PLoS One. 2022 Apr 14;17(4):e0266007. doi: 10.1371/journal.pone.0266007 (PMC9009705; doi:10.1371/journal.pone.0266007)
Supplement: Attachment — Submitted filename: Response to reviewers.docx [file pone.0266007.s005.docx]

Dear Richard Bruce Mink

On behalf of all the authors, I thank you for your interest in our manuscript *“We can’t do without it”: parent and call-handler experiences of video triage of children at a medical helpline*, PONE-D-21-08760.

We are today submitting a revised version, changed according to your points raised. Consequently, this manuscript now contains all data concerning the users’ experiences of video triage studies conducted at the Medical Helpline 1813, whereas the accompanying paper *(Video triage of children with respiratory symptoms at a medical helpline is safe and feasible – a prospective quality improvement study,* PONE-D-21-06235) solely focuses on patient outcome.

We have also revised the manuscript’s set-up, so it conforms with the style requirements. Furthermore, ast the sharing of sensitive patient data originating from quality improvement projects is not permitted by Danish law, anonymized minimal data sets have been prepared and are accessible on reasonable requests to the contact person mentioned in the manuscript.

This manuscript now conveys quite a rich picture of how the contemporary tool video streaming was received and experienced by the users, when being implemented at a functioning, busy call-center. Furthermore, the sub-study on project evaluation may aid other call-centers who are considering the implementation of a similar tool. We sincerely hope that the papers are accepted, as we believe that that may contribute with new and important results regarding health services, relevant to many health care providers globally.

Kind regards,

Dr. Caroline Gren
